# Assessment of various veterinary drug residues in animal originated food products

**DOI:** 10.14202/vetworld.2021.1650-1664

**Published:** 2021-06-28

**Authors:** Jagdish Kumar Parmar, Kundan Kumar Chaubey, Vikas Gupta, Manthena Nava Bharath

**Affiliations:** 1 Department of Biotechnology, GLA University, Chaumuhan, Mathura, Uttar Pradesh, India; 2 TUV India Pvt. Ltd., Sus Rd, Mulshi, Pune, Maharashtra, India; 3 EUREKA Analytical Services Pvt. Ltd. 31 Milestone, Main GT Road, Kundli, Sonepat, Haryana, India

**Keywords:** antibacterial, antibiotics, food products, methods, techniques, veterinary drug residues

## Abstract

The veterinary drugs are broad-spectrum antibacterial antibiotics; it uses to cure the animal disease. Many countries have banned veterinary drug residues like nitrofurans metabolites, chloramphenicol. However, the people were administrated veterinary drugs to animals as illegal to increase the milk production in animals for economic benefit. The results of illegally use of veterinary drugs remain as a residue in animal product like milk and it is very harmful to whom consume it cause cancer and allergic for human being which has entered the concern among milk consumers. To control illegal use of veterinary drugs, the government of India has restricted its use in animals. For the identification and confirmation of veterinary drug residues in animal products, analytical techniques such as liquid chromatography and mass spectrometry are available. These are very sophisticated equipments which are available nowadays and their methodologies for the analytical method validation are described by European commission 2002/657/EC. The use of veterinary drugs is a big challenge to effectively identify and authorization of their use. There are so many analytical techniques are using very effectively and taking very less time to protect the consumers from their adverse effects. These techniques take very less time to identify more groups of compounds such as tetracycline, sulfonamides, anthelmintic, and macrolides in single multi-residue method. These methods having validation parameters include system precision, calibration curve, accuracy, limit of detection, and quantification. Therefore, improvement in the existing technologies and accessibility of new screening methodologies will give opportunities for automation that helps in obtaining the results in very less time and improved sensitivity and specificity which contribute to better safety assurance, standard, and quality of various food products of animal origin.

## Introduction

The veterinary drugs are used in very large amounts to cure animal disease as well as inhibit bacterial growth in animals. The study of the antimicrobials/drug residues in various foods that originated from animals began in late 1960 and early 1970s in most of the European countries, for example: Belgium, Netherlands, and Luxembourg [1]. The use of antimicrobials/drugs in animals producing milk and meat may leave their residues in the foodstuffs due to illegal use of unlicensed antibiotics, extra-label of their doses, failure to observe the period of withdrawal of drugs, and contamination of the animal feeds with the excreta of drugs/antimicrobials treated animals [2]. The banned antibiotic residues use in food-producing animals is an illegal activity because residues remain in byproducts of animals (milk and meat) which gives adverse effects to consumers [2]. Various investigations pointed out the consumption of byproducts from these animals are the major source of human inadvertent antibiotic intake [3]. Antibacterial drugs have used diverse areas of animal farming as therapeutic agents against different pathogenic microbes [4]. We all are well known about cow’s milk which contains a good balance of proteins, fats, and carbohydrates. It is an indispensable food because it is inexpensive and easily available [5]. In the current scenario, dairy products have their unique properties, such as sheep and goat milk (in goat milk, better ratios of amino acids and milk protein are easily digested and more in nutritional benefit). In goat and sheep milk aS1 fraction are absent, which causes celiac disease in infants [6]. There are so many powerful analytical techniques are available to estimate the food parameters for antibiotics residues, such as liquid chromatography-tandem mass spectrometry (LC-MS/MS) and high-pressure LC (HPLC) with a different type of detector as per application or nature of the analytes and Enzyme-Linked immunosorbent assay (ELISA) [7]. The estimation of veterinary drug residues can be done through HPLC but there are some limitations we cannot go very low level as per mention in regulation especially for minimum required performance limit (MRPL) compound such as Chloramphenicol (Chl^+^), Nitrofurans metabolites only minimum required limit compound can be quantified through HPLC. There are major drawbacks of HPLC; it takes more time for quantification through instrument as well as a different group of compounds also not possible through it. It required a different method for quantification for a different group. It is a time taking process; nowadays there are some more sophisticated techniques like LC-MS/MS is using. This technique is very less time taking and the quantification of the number of compounds as well as groups is very easy. It is a very effective technique for MRPL compound that there is a limit of quantitation (LOQ) is very low. The analysis of veterinary drug residues with a different group of antibiotic residues in a single multi-residue method is possible through LC-MS/MS. There are major advantages of LC-MS/MS its gives quantitative as well as confirmative analysis at very low levels [8].

## Analytical Techniques in Antibiotics Residues Analysis

The analytical method has multiple techniques to identify and quantify the antibiotic residues in different various food matrixes. The identification of these drug molecules is based on microbial technique like ELISA. The quantification method is based on m/z (mass to charge ratio) of the compounds and RT of the compounds with conformation of daughter products with ion ratio of the compounds. Different type of chromatographic method is available such as LC using ultraviolet detector (UV), diode array detection (DAD), and fluorescence detection (FLD).

Analytical techniques such as ELISA, HPLC, and LC-MS/MS are very important in identification, quantification, and confirmation of veterinary drug residues that contribute to a better safety guarantee, standard, and quality of various animal origin food products (Tables-1,2 and 3).

**Table-1:** Recommended maximum residue limit (MRL) for antibiotics in milk as per Food Safety and Standards Authority of India (FSSAI).

Name of the antibiotics	FSSAI MRL mg/kg milk (ppm)
Thiabendazole	0.100
Praziquantel	0.010
Fenbantel	0.01
Monensin	0.002
Parbendazole	0.010
Diminazene	0.010
Lincomycin	0.150
Tet^+^	0.100
Chlortet^+^	0.100
Oxytet^+^	0.100
Ceftiofur	0.100
Tylosin	0.100
Virginiamycin S1	0.010
Virginiamycin M1	0.010
Doramectin	0.010
Flunixin	0.010
Trimethoprim	0.010
Sulfadimidine	0.010
Sulfaquinoxaline	0.010
Albendazole	0.100
Fenbendazole	0.010
Oxfendazole	0.010
Ampicillin	0.010
Ivermectin	0.010

**Table-2:** Recommended maximum residue limit for antibiotics in various matrix as per FSSAI.

Sr. No.	Name of compounds	Matrix	FSSAI MRL (mg/kg)
1.	Amp^+^	Milk	0.01
		Animal tissues (Edible)	0.01
		Fats (animal tissue)	0.01
2.	Cloxacillin	Animal tissues (Edible)	0.01
		Fats (animals)	0.01
		Milk	0.01
		Tissues	0.01
3.	Chlor-tet^+^ Oxy-tet^+^/ tet^+^	Cattle milk	0.1
		Muscle (tissue)	0.2
		Kidney (tissue)	1.2
		Liver (Tissue)	0.6
		Giant prawn (Paeneus monodom muscle)	0.2
		Pig: Muscle (Tissue)	0.2
		Kidney (Tissue)	1.2
		Liver (Tissue)	0.6
		Poultry: Kidney (Tissue)	1.2
		Liver (Tissue)	0.6
		Muscle (Tissue)	0.2
		Sheep: Muscle (Tissue)	0.2
		Milk	0.1
		Liver (Tissue)	0.6
		Kidney (Tissue)	1.2
4.	Erythromycin	Chicken: Liver (Tissue)	0.1
		Kidney (Tissue)	0.1
		Eggs	0.05
		Fat	0.1
		Turkey: Liver (Tissue)	0.1
		Muscle (Tissue)	0.1
		Fat	0.1
		Kidney (Tissue)	0.1
5.	Flumequine	Cattle: Fat	1
		Muscle (Tissue)	0.5
		Kidney (Tissue)	3
		Liver (Tissue)	0.5
		Chicken: Fat	1
		Liver (Tissue)	0.5
		Kidney (Tissue)	3
		Muscle (Tissue)	0.5
		Sheep: Fat	1
		Kidney (Tissue)	3
		Liver (Tissue)	0.5
		Muscle (Tissue)	0.5
		Trout: Muscles (Tissue)	0.5
		Pig: Fat	1.0
		Kidney (Tissue)	3
		Muscle (Tissue)	0.5
		Liver (Tissue)	0.5
6.	Lincomycin	Chicken: Muscles (Tissue)	0.2
		Fat	0.1
		Liver (Tissue)	0.5
		Kidney (Tissue)	0.5
		Cattle: Milk	0.15
		Pig: Liver (Tissue)	0.5
		Kidney (Tissue)	1.5
		Muscle (Tissue)	0.2
		Fat	0.1
7.	Neomycin	Cattle: Liver (Tissue)	0.5
		Muscle (Tissue)	0.5
		Kidney (Tissue)	10
		Fat	0.5
		Milk	1.5
		Chicken: Liver (Tissue)	0.5
		Fat	0.5
		Muscle (Tissue)	0.5
		Kidney (Tissue)	10
		Eggs	0.5
		Duck: Fat	0.5
		Muscle (Tissue)	0.5
		Liver (Tissue)	0.5
		Kidney (Tissue)	10
		Sheep: Muscle (Tissue)	0.5
		Fat	0.5
		Liver (Tissue)	0.5
		Kidney (Tissue)	10
		Trout (Muscle)	0.5
		Pig: Liver (Tissue)	0.5
		Kidney (Tissue)	10
		Muscle (Tissue)	0.5
		Fat	0.5
		Goat: Liver (Tissue)	0.5
		Kidney (Tissue)	10
		Muscle (Tissue)	0.5
		Fat	0.5
		Turkey: Liver (Tissue)	0.5
		Kidney (Tissue)	10
		Muscle (Tissue)	0.5
		Fat	0.5
8.	Spectinomycin	Cattle: Muscle (Tissue)	0.5
		Liver (Tissue)	2
		Kidney (Tissue)	5
		Fat	2
		Milk	0.2
		Chicken: Muscle (Tissue)	0.5
		Liver (Tissue)	2
		Kidney (Tissue)	5
		Fat	2
		Eggs	2
		Pig: Muscles (Tissue)	0.5
		Liver (Tissue)	2
		Kidney (Tissue)	5
		Fat (Tissue)	2
		Sheep: Muscle (Tissue)	0.5
		Liver (Tissue)	2
		Kidney (Tissue)	5
		Fat	2
9.	Trimethoprim	Animal tissues (Edible)	0.01
		Fats (animal tissues)	0.01
		Milk	0.01
10.	Sulfadiazine	Animal tissues (Edible)	0.01
		Fats (animal tissues)	0.01
		Milk	0.01
11.	Sulfanilamide	Animal tissues (Edible)	0.01
		Fats (animal tissues)	0.01
		Milk	0.01
12.	Zinc Bacitracin	Animal tissues (Edible)	0.01
		Fats (animal tissues)	0.01
		Milk	0.01
13.	Amprolium	Animal tissues (Edible)	0.01
		Fats (animal tissues)	0.01
		Milk	0.01
14.	Apramycin	Animal tissues (Edible)	0.01
		Fats (animal tissues)	0.01
		Milk	0.01
15.	Ceftiofur	Cattle: Liver (tissue)	2
		Kidney (tissue)	6
		Milk (tissue)	0.1
		Fat (tissue)	2
		Muscle (tissue)	1
		Pig: Muscles (tissue)	1
		Liver (tissue)	2
		Kidney (tissue)	6
		Fat	2
16.	Cephapirine	Animal tissues (Edible)	0.01
		Fats (animal tissues)	0.01
		Milk	0.01
17.	Clopidol	Animal tissues (Edible)	0.01
		Fats (animal tissues)	0.01
		Milk	0.01
18.	Danofloxacin	Cattle: Liver (tissue)	0.4
		Kidney (tissue)	0.4
		Fat	0.1
		Muscle (tissue)	0.2
		Pig: Muscles (tissue)	0.1
		Liver (tissue)	0.05
		Kidney (tissue)	0.2
		Fat	0.1
		Chicken: Muscles (tissue)	0.2
		Liver (tissue)	0.4
		Kidney (tissue)	0.4
		Fat	0.1
19.	Nicarbazin	Chicken: Kidney (tissue)	0.2
		Fat/Skin (tissue)	0.2
		Liver (tissue)	0.2
		Muscle (tissue)	0.2
20.	Monensin	Cattle: Liver (tissue)	0.1
		Muscle (tissue)	0.01
		Kidney (tissue)	0.01
		Fat	0.1
		Milk	0.002
		Sheep: Muscle (tissue)	0.01
		Liver (tissue)	0.02
		Kidney (tissue)	0.01
		Fat	0.1
		Goat: Liver (tissue)	0.02
		Muscle (tissue)	0.01
		Kidney (tissue)	0.01
		Fat	0.1
		Chicken: Kidney (tissue)	0.01
		Muscle (tissue)	0.01
		Liver (tissue)	0.01
		Fat	0.1
		Turkey: muscle (tissue)	0.01
		Liver (tissue)	0.01
		Kidney (tissue)	0.01
		Fat	0.1
		Quail: Liver (tissue)	0.01
		Kidney (tissue)	0.01
		Muscle (tissue)	0.01
		Fat	0.1
21.	Moxidectin	Cattle: muscles (tissue)	0.02
		Liver (tissue)	0.1
		Kidney (tissue)	0.05
		Fat	0.5
		Sheep: Muscle (tissue)	0.05
		Liver (tissue)	0.1
		Kidney (tissue)	0.05
		Fat	0.5
22.	Sulfaquinoxaline	Animal tissues (Edible)	0.01
		Fats (animal tissues)	0.01
		Milk	0.01
23.	Sulfadimidine (Sulfamethazine)	Cattle: Milk	0.01
		Muscle (tissue)	0.1
		Fat	0.1
		Kidney (tissue)	0.1
		Liver (tissue)	0.1
24.	Tylosin	Cattle: muscles (tissue)	0.1
		Liver (tissue)	0.1
		Kidney (tissue)	0.1
		Fat	0.1
		Pig: Muscle (tissue)	0.1
		Liver (tissue)	0.1
		Kidney (tissue)	0.1
		Fat	0.1
		Sheep: Muscle (tissue)	0.1
		Liver (tissue)	0.1
		Kidney (tissue)	0.1
		Chicken: Muscle (tissue)	0.1
		Liver (tissue)	0.1
		Kidney (tissue)	0.1
		Fat/Skin (tissue)	0.1
		Eggs	0.3
25.	Virginiamycin	Animal tissues (Edible)	0.01
		Fats (animal tissues)	0.01
		Milk	0.01
26.	Albendazole	Muscle (tissue)	0.1
		Liver (tissue)	5
		Kidney (tissue)	5
		Fat	0.1
		Milk	0.1
27.	Closantel	Cattle: muscle (tissue)	1
		Liver (tissue)	1
		Kidney (tissue)	3
		Fat	3
		Sheep: muscles (tissue)	1.5
		Liver (tissue)	1.5
		Kidney (tissue)	5
		Fat	2
28.	Doramectin	Cattle: muscle (tissue)	0.01
		Liver (tissue)	0.1
		Kidney (tissue)	0.03
		Fat	0.15
		Milk	0.015
		Pig: Muscles (tissue)	0.005
		Liver (tissue)	0.1
		Kidney (tissue)	0.03
		Fat	0.15
29.	Flunixin	Animal tissues (Edible)	0.01
		Fats (animal tissues)	0.01
		Milk	0.01
30.	Ivermectin	Milk	0.01
		Liver (tissue)	0.8
		Fat	0.4
		Muscle (tissue)	0.03
		Kidney (tissue)	0.1
		Pig: Liver (tissue)	0.015
		Fat	0.02
		Sheep: Liver (tissue)	0.015
		Fat	0.02
31.	Levamisole	Cattle: muscles (tissue)	0.01
		Liver (tissue)	0.1
		Kidney (tissue)	0.01
		Fat (tissue)	0.01
		Sheep: Muscles (tissue)	0.01
		Liver (tissue)	0.1
		Kidney (tissue)	0.01
		Fat	0.01
		Poultry: Muscle (tissue)	0.01
		Liver (tissue)	0.1
		Kidney (tissue)	0.01
32.	Meloxicam	Animal tissues (Edible)	0.01
		Fats (animal tissues)	0.01
		Milk	0.01
33.	Oxfendazole	Animal tissues (Edible)	0.01
		Fats (animal tissues)	0.01
		Milk	0.01
34.	Febantel/Fenbendazole/Oxfendazole	Cattle: Muscle (tissue)	0.1
		Liver (tissue)	0.5
		Kidney (tissue)	0.1
		Fat	0.1
	Oxyclozanide	Milk	0.1
		Pig: Muscle (tissue)	0.1
		Liver (tissue)	0.5
		Kidney (tissue)	0.1
		Fat	0.1
		Sheep: Muscles (tissue)	0.1
		Liver (tissue)	0.5
		Kidney (tissue)	0.1
		Fat	0.1
		Milk	0.1
		Goat: Liver (tissue)	0.5
		Muscle (tissue)	0.1
		Kidney (tissue)	0.1
		Fat	0.1
35.		Animal tissues (Edible)	0.01
		Fats (animal tissues)	0.01
		Milk	0.01
36.	Parbendazole	Animal tissues (Edible)	0.01
		Fats (animal tissues)	0.01
		Milk	0.01
37.	Praziquantel	Animal tissues (Edible)	0.01
		Fats (animal tissues)	0.01
		Milk	0.01
38.	Sulfa Chloropyrazine	Animal tissues (Edible)	0.01
		Fats (animal tissues)	0.01
		Milk	0.01
39.	Thiabendazole	Cattle: Muscles (tissue)	0.1
		Liver (tissue)	0.1
		Kidney (tissue)	0.1
		Fat	0.1
		Milk	0.1
		Pig: Muscle (tissue)	0.1
		Liver (tissue)	0.1
		Kidney (tissue)	0.1
		Fat	0.1
		Sheep: Muscles (tissue)	0.1
		Liver (tissue)	0.1
		Kidney (tissue)	0.1
		Fat	0.1
		Goat: Liver (tissue)	0.1
		Muscle (tissue)	0.1
		Kidney (tissue)	0.1
		Fat	0.1
		Milk	0.1
40.	Triclabendazole	Cattle: Muscles (tissue)	0.25
		Liver (tissue)	0.85
		Kidney (tissue)	0.4
		Fat/Skin (tissue)	0.1
		Sheep: Muscle (tissue)	0.2
		Liver (tissue)	0.3
		Kidney (tissue)	0.2
		Fat/Skin (tissue)	0.1
41.	Xylazine	Animal tissues (Edible)	0.01
		Fats (animal tissues)	0.01
		Milk	0.01
42.	Diminazene	Cattle: Muscles (tissue)	0.5
		Liver (tissue)	12
		Kidney (tissue)	6
		Milk	0.15
43.	Cefacetrile	Animal tissues (Edible)	0.01
		Fats (animal tissues)	0.01
		Milk	0.01

**Table-3:** Recommended MRL for antibiotics in honey as per food safety and standards authority of India FSSAI.

Sr. no.	Name of compounds	Matrix	Maximum residue limit (MRL)
1	Chl^+^	Honey	≤0.3 µg/kg
2	Nitrofurans and its metabolites	Honey	≤0.5 µg/kg either individually or collectively
3	Sulfa^+^ group	Honey	≤5.0 µg/kg either individually or collectively
4	Streptomycin	Honey	≤5.0 µg/kg
5	Tet^+^	Honey	≤5.0 µg/kg
6	Oxy-tet^+^	Honey	≤5.0 µg/kg
7	Chlor-tet^+^	Honey	≤5.0 µg/kg
8	Ampicillin	Honey	≤5.0 µg/kg
9	Enrofloxacin	Honey	≤5.0 µg/kg
10	Cipro^+^	Honey	≤5.0 µg/kg
11	Erythromycin	Honey	≤5.0 µg/kg
12	Tylosin	Honey	≤5.0 µg/kg

## ELISA

In 1971, the technique was 1^st^ time explained by Engvall and Perelman. It is a very common and ancient technique to use in medicine, plant pathology, biochemistry, and biotechnology as a diagnostic tool. The principle of ELISA is based on antigen and antibody reaction. The final results observe as a color change through reaction (Figure-1). Antigen is attached on a sample surface as adsorption so it’s easily attached with same specific antibody. The ELISA technique is also called semi-quantitative technique because it works quantitatively as well as qualitatively. In a study, 27 antibiotics residue analyzed in cow’s milk and milk products. In this method, ELISA used as a screening method based on enzyme-linked tool for qualitative analysis to identify the antibiotic residues in different types of milk products in a single test. Method validation as per European Community Reference Laboratory (ECRLs) guidelines with method criteria evaluated including rate, false-negative (FN), false-positive (FP) rate, and detection capability. As EC regulation EC/37/2010 out of 27 antibiotics, four antibiotics (Tet^+^, Oxy-tetracycline [Oxy-tet^+^], nafcillin and rifaximin) showed false-negative rate ranging between 1.7 and 4.9% [9]. A comparison study done in Guelma’s farms (Algeria) in raw and fermented cow’s milk. The first method done by delvotest SP-NT in this study the false-negative results is very high so it’s less trustworthy. The second method confirmation by LC-MS/MS with trace of antibiotics in numerous samples found antibiotics residues suggested a lack of public health control as well in livestock industry [10].

**Figure- 1:**
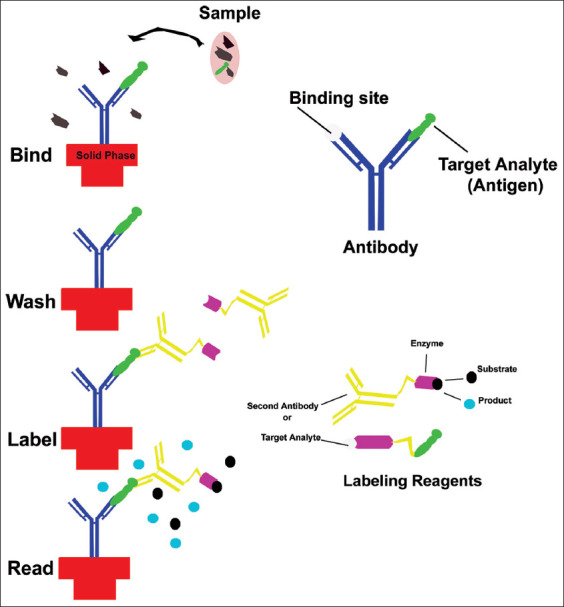
Illustration of enzyme-linked immunosorbent assay [Source: Figure prepared by Manthena Nava Bharath].

## HPLC

**Figure-2:**
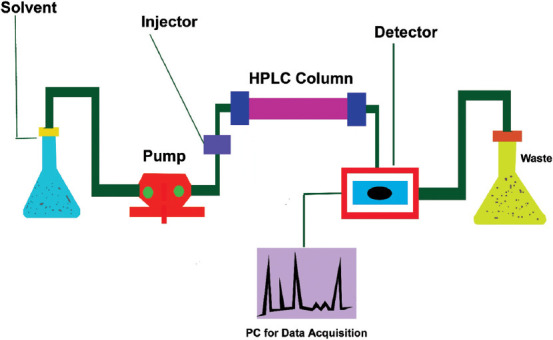
Flow diagram of high-pressure liquid chromatography [Source: Figure prepared by Manthena Nava Bharath].

It is also called as HPLC technique because solvent push through the pump pressurization. Basically, it’s divided into five major parts first mobile phase, second detector, third pump (binary and quaternary), fourth column oven, and fifth autosampler. Each and every component of HPLC shows its specific features. In HPLC, previously samples kept in auto-sampler where it’s come through the injector and carry through the mobile phase flow which it reaches into the column. The pumps generate optimum flow, pressure, and composition of the mobile phase through the column thereafter the sample passed through the column and reaches into a detector where it generate a signal proportional to the amount of sample it comes as quantitative analysis of the sample component in the samples. Basically, two techniques are involved in HPLC, first normal phase second reverse phase. In normal phase using mobile phase a form of non-polar work as while stationary phase work as polar. In reverse phase technology, stationary phase and mobile phase work as a non-polar and polar form, respectively. There is one important role have in pump like mobile phase composition mixing through pump (i) iso crating- pre-set mobile there is no any change require during the entire mobile phase in any type of method so pressure seems to be observed as a constant flow (ii) gradient-in this the mobile composition can be changed parallelly with time. It depends on the optimized method during the development of a particular analyte. Hence, the chances of pressure fluctuation seem to be more that is sometimes high and sometimes low. There are many types of columns available in markets with different make; column is filled with silica as SIO_2_ form a column pressure also vary use particle size of silica, smaller particle size packing generates high pressure while larger particle size generates low pressure. The mobile phase, as usual based on composition of two phase’s aqueous and organic phases. In aqueous using salt such as K_2_HPO_4_, TEA, and NAH_2_PO_4_ dissolved in water however, in organic phases commonly use acetonitrile, methanol, toluene, etc. There are many types of detectors use in HPLC (1) UV (UV/visual, 200-400 for UV and 400-800 for visual), (2) photodiode array (PDA), (3) refractive index (RI) detector, and (4) fluorescence detector (FD). The use of detector based on required parameters such as sugar profile quantification through RI, aflatoxins through FD, and mostly parameters quantification come through PDA and UV/VIS (Figure-2). The total analytical time was <13 min. Range of testing was 0.05-2.0 mg/mL and recovery from 85.3 to 90.6% [5].

The effect of methodology of cooking of ten samples with frying, boiling, and grilling of chicken muscle had reduced the concentration of Oxy-tet^+^ of 84.52-96.58% and for Amp^+^ residues, it reduced with 81.22, 90.54, and 94.5% after frying, boiling, and grilling, respectively. The obtained results allow to conclude that the examined sample collected from local and frozen imported broilers giblets and tissue proved to be contaminated with residues of Oxy-tet^+^ and Amp^+^ especially in liver sample [2]. Romero et al. [11] developed and described the method which concluded the effect of albendazole in goat milk and microbial inhibitor used of screening of antibiotics were evaluated with total of eighteen goats (Murciano-Granadina) treated with albendazole and collected milk from them after 7 days. HPLC is a technique used for identification and quantification of the albendazole and results found the albendazole was not detected but the metabolite of the same was found 1-day post-treatment lower than the Maximum Residual Limit (MRL) 100 μg/kg. Kim et al. [12] developed and validated the HPLC with a detector of PDA and sample extraction by immune-affinity column chromatography. In this method, total 16 Sulfa^+^ residues were determined and validation was done includes linearity, selectivity, sensitivity, precision, and accuracy of the test. The limit of detection (LOD) of the method was 14.1-45 μg/kg and the average recovery of the same ranged 78.2-105.25% and interday and intraday precision was <5.5%. Patyra et al. [13] validated a non-targeted feed sample method as per EC 657/2002 in Sulfa^+^ by HPLC with FLD and sample extraction with pre-column derivatization. The method parameters are selectivity, detection capability and decision limit, linearity, accuracy, and precision. In this method, recovery was 79.3-114%, decision limit was 197.7-274.6 μg/kg, and detection capability was 263.2-337.9 μg/kg, repeatability was 2.7-9.1%, and reproductivity with 5.9-14.9% depending on the analytes. Kellnerová et al. [14] measured the effect of spiked study with Tet^+^ and Oxy-tet^+^ in high pasteurization (85°C/3 s) of raw cow milk and measure after and before heating whereas the residue of the tet+ and oxy-tet+ was decreased 5.74% and 15.3%, respectively. Saleh et al. [15] extracted Tet^+^ and Oxy-tet^+^ residues from honey samples purchased from local market. The method of sample extraction used for honey sample was 0.01M sodium succinate butter and the examined sample had 31.25% and 12.50% Oxy-tet^+^ and tet^+^ residues, respectively, and the quantification of the sample by HPLC with UV detector. Chauhan et al. [16] used different validation parameters such as LOD, system suitability, specificity, precision and accuracy, linearity, and limit of quantification. The LOQ was 98, 94, and 23 μg/kg. LOD were 48, 44, and 8mg/kg, for Oxy-tet^+^, Tet^+^, and Chlor-tet^+^, respectively, and average recovery found between 71 and 110%. Marinou et al. [17] developed and validated an HPLC as per European Union Decision 2002/657/EC with DAD using a simple sample extraction procedure with acetonitrile as an extraction solvent and for clean-up C18 and PSA are used in Tet^+^, Oxy-tet^+^, and Chlor-tet^+^ in milk sample. The method was linearity, ruggedness, selectivity, precision and accuracy, and sensitivity. The recoveries were in between 83.07% and 106.3% at 100 and 200 μg/kg, detection capability was 100.6 and 109.7 μg/kg, and decision limit was 100.3 and 105.6 μg/kg. Another method was used for determination by HPLC with UV-Visible detector of Tet^+^, Oxy-tet^+^, chlor-tet^+^, and doxycycline in pasteurized cow milk. The sample extraction was based on the deproteinization, extraction as well as clean-up by solid phase, which was validated as per EC/657/2002. The precision and accuracy were performed at level of 0.5, 1.0, and 1.5 times the maximum permissible limit allowed in Brazil and the detection limit and decision capability were 129.3-188.7 and 114.2-143.7, respectively; the recovery was 82.5-114.5% with a precision of 7.1% [18]. An additional method was performed at Egypt and Giza governorate, 50 random samples were collected from fresh beef market. The results showed the incidence of antibiotics in examined beef samples; they found two samples have β-lactam Penicillin (β-lac^+^), one sample has Oxy-tet^+^, three samples have aminoglycosides, and one sample has Cipro+, respectively. Findings showed that 2% samples contain β-lac^+^ + Sulfonamides (Sulfa^+^) and Ciprofloxacin (Cipro^+^) + Sulpha^+^ + Oxy-tet^+^, Oxy-tet^+^, β-lac^+^ + Aminoglycoside + Sulfa^+^, Cipro^+^, Macrolides + Aminoglycoside + Cipro^+^, β-lac^+^, Macrolides + β-lac^+^, Macrolides + Oxy-tet^+^, and 4% of samples for Oxy-tet^+^ + Aminoglycoside + Macrolides + Cipro^+^. This study was performed in 13 samples of beef out of 50 available samples. It was found that after boiling for 30 min, reduction in Cipro+ and Oxy-tet+ residues was measured by 20.74% and 87.97%, respectively. In microwave after 20 min incubation there was reduction of Cipro^+^ and Oxy-tet^+^ residues was measured by 38.14% and 86.95%, respectively. Determination of Cipro^+^ and Oxy-tet^+^ residues was measured in experimentally treated rabbits’ samples (50 rabbit) after cooking (boiling, microwave, and roasting) and freezing treatment. Microwaving, boiling, and roasting are the three more effective heat treatment methods than roasting and freezing. Shaltout et al. [19] used HPLC for the determination of Cipro^+^. They incubated the samples by freezing and found 65.73% and 100.0% reduction in 6 months and 12 months. They had also determined the reduction of Oxy-tet^+^ residues at freezing temperature and found 4.41% and 30.01% reduction in 6 and 12-months, respectively. Cipro^+^ residues are heat stable so; microwave and freezing methods are only effective to degrade Cipro^+^ residues to a safety level. Mostafa et al. [20] used UPLC/UV and LC-MS/MS to determine an antibiotics residue in fish muscle and water samples as per the ICH guidelines. The veterinary drugs include florfenicol, sulfadiazine, flumequine, nalidixic acid, trimethoprim, doxycycline, Chlor-tet^+^, and sulfathiazole. The sample extraction of the method was simple as solid-phase extraction. The limit of detection and limit of quantifications of the method are 0.2-0.4 and 0.3-0.6 μg/kg in fish muscle, respectively, and 0.005-0.02 and 0.01-0.08 μg/mL in water, respectively. Darko et al. [21] analyzed traces of antibiotics/drugs residues in dairy products having adverse on human consumption in Kumasi, Ghana. Common veterinary drug residues used in their countries were sulfathiazole, Chl^+^, Oxy-tet^+^, and sulfamethoxazole. They estimated the recovery range 78-97% and linearity correlation coefficient was ≥0.9991, the highest recovery for Chl^+^ was 97% and least recovery of 78% was in sulfamethoxazole, and the LOD was 0.1 μg/kg for Chl^+^. Kurjogi et al. [22] evaluated the impact of factors like pH and temperature on the stabilities of antibiotics (azithromycin and Tet^+^) by HPLC in Karnataka, India. They reported a significant reduction in stability and antimicrobial activity of solution of Tet^+^ and azithromycin when incubated for 24 h at 70 and 100°C and at acidic pH 4-5. They have detected both of the antibiotics in cow milk at high concentrations of 9708.7 and 5460 μg/kg, respectively. Orwa et al. [23] investigated the occurrence of thirteen veterinary drug residues of Tet^+^ and Sulfa^+^ along the dairy sub-value chain in Nakuru country. Samples were screened using Charm II Blue-Yellow-test and for confirmation, they used HPLC UV detector for sulfa-chloro-pyridazine, sulfadiazine, sulfadimidine, sulfaquinoxaline, sulfamerazine, sulfathiazole, sulfamethoxazole, sulfadoxine, sulfadimethoxine, Oxy-tet^+^, doxycycline-hyclate, Chlor-tet^+^ hydrochloride, and Tet+ hydrochloride. They found 31.4% (72/229) and 28.8% (23/80) samples were found positive for antimicrobial residues collected from rural and peri-urban backgrounds, respectively.

## LC-MS

The LC tandem MS (LC-MS/MS) is a highly sophisticated technique. The principle of the LC is separation of ion based on polarity with the help of stationary phase as well mobile phase like column and the MS is a work based on mass to charge ratio (M/Z Ratio) of the component to be identified and quantification. In LC-MS/MS technique, there is ionization source such as electron spray ionization (ESI), atmospheric pressure chemical ionization (APCI), atmospheric pressure photoionization (APPI) which are based on application and nature of the compounds like-polar in nature, mid-polar, and non-polar. It is giving quantitative results with a known concentration of linear standard calibration curve. In LC-MS/MS technique, triple quadrupole consist of four parallel rods with different charges and its will changes between the traveling of the ions because of radio frequency (RF) voltages and its gives helical path to the ion travelers in a free atmosphere pressure to reach the detector, first quadrupole identified mass of the parent compound molecules, and second quadrupole works like collision cell its fragments parent compound into different masses with the help of argon gas based on week bond of the compound and third quadrupole works for identification and quantification of the fragmented ion called as daughter ion. It gives confirmation of the particular analyte with two different fragmented ions. The MS detector is classified into i. electron multiplier detector and Photon multiplier detector- providing a current output proportional to light intensity. Photomultipliers are used to measure any process which directly or indirectly emits light. (Figure-4). The confirmation of molecules is based on the retention time (RT) of the analytes in the reference standard as well as in the samples. RT is the time where the analytes is eluent from the column its based on nature of component likes – polar, non-polar and mid polar, and ion ratio of the analytes as well as sample in the different matrix, the ion ratio is the ratio of qualifier ions versus quantifier ions. In this method, they used porous aromatic framework based solid-phase extraction for the extraction of macrolide antibiotics in chicken samples with comparison of traditional solid-phase extraction. It showed good adsorption capacity and reproducibility. LOD was estimated in range 0.2-0.5 μg/kg and recovery range 82.1-101.4% with relative standard deviation (RSD) <11.1% [3]. Maddaleno *et al*. [4] reported the LOD was 19, 22, and 10 μg/kg and limit of quantification was 62, 73, and 34 μg/kg in feather, muscle, and liver, respectively. Recovery levels reported between 98 and 101% and calibration curve (r²) reported >0.99. Tao *et al*. [7] did Matrix solid-phase dispersion with quantitative and qualitative analysis using LC-ESI-MS/MS. They reported that LOQ was 25 μg/kg in feed sample and recovery of the spike concentration range 25-100 μg/kg was found 75.9-87.9%. Hamamoto and Mizuno [24] did a study on ten males and ten females white Leghorn chicken for a week related to Amp+ concentrations of liver, kidney, and skin at 2 days withdrawal. Chickens fed the diet containing β-lac^+^ 40 mg/kg per kg body weight/day for a week. LC-MS/MS method used for measuring the Amp^+^ residue and the mean recoveries and LOQ was 93-102.7% and 0.1-1.4 ng/g, respectively. Jedziniak *et al*. determined wide range of residues of four metamizole metabolites, five corticosteroids, and 16 anti-inflammatory drugs in minced muscle samples. The method validated as per commission decision 2002/657/EC detection capability (CC-β), linearity, decision limit CC-α, precision, and accuracy and were calculated using LC-MS/MS [25]. Chen *et al*. [26] determined the flunixin residues in foods of animal origin which pose hazards for human health. They analyzed 5-hydroxyflunixin residue in milk and flunixin residue in bovine muscle by ELISA and validated by LC-MS/MS confirmatory method with coefficient variation ranging 5.8-11.3% and average spike recovery ranged 83-105%. Jedziniak *et al*. [6] have analyzed 17 veterinary drugs such as Sulfa+, macrolides, and anthelmintic by LC-MS/MS in sheep-goat milk and dairy products from polish market. They detected very small percentage (0.83%) of drug residues. All samples found passed (CC-α 1-10 μg/kg) except one sample of cottage cheese. ABZ sulfone was detected 5.2 μg/kg and confirmed, thiabendazole was detected in two cheese samples trace around 0.7 μg/kg. Unsal *et al*. [27] have developed and validated isotope-dilution LC with MS with heated ESI Source method for estimation of Sulfa^+^ in 14 milk samples obtained from market and street vendors. Recovery results were found between 91 and 114% and relative measurement uncertainty was measured between 7.5 and 12.7%. The concentration of Sulfa^+^ found below the legal limits, for example, sulfamethazine: 6.46±0.76ng/g and sulfisoxazole: 7.3±0.71ng/g. A sensitive and selective method was developed and validated was positive ion LC-MS method of 10 sulfonamides antibiotic residue in raw shrimp meat as per EU guidelines council Directive 2002/657/EC. Method validation parameters are system suitability % RSD range from 10.90 to 18.58%, specificity average area of blank sample was in range from 24 to 273, LOD was in range from 7.2 to 17.7, LOQ was in range from 21.7 to 53.5, recovery at 50ppb level in range between 87.39 and 101.87%, at 100ppb level range between 87.14 and 101.69%, at 150ppb level range between 97.41 and 106.35% with three batches, DL (CCα) were range from 104.72 to 106.60%, DC (CCβ) ranged between 114.33 and 117.98, and for ruggedness % RSD range between 3.11 and 5.19% [8]. Wang *et al*. [28] evaluated and compared three methods for extraction of Sulfa^+^ in porcine tissues. These three methods are (1) Oasis PRiME hydrophillic-lipophilic balance (HLB), (2) Enhanced Matrix Removal for Lipid (EMR-L), and (3) conventional liquid-liquid extraction with n-hexane (LLE) sample preparation. They analyzed the samples by ultra HPLC (UHPLC) and detected by a triple quadrupole MS/MS or a quadrupole-time-of-flight tandem MS (Q-TOF/MS). Matrix effect from EMR-L and HLB was significantly lower than that from LLE. Recoveries were quantified for 17 samples for the determination of Sulfa^+^ by the matrix-matched calibration curve at spiked level of 5, 10, and 20 μg/kg, and results were 97.0%. Govind *et al*. [29] have developed and validated LC-MS/MS in accordance with European commission 2002/657/EC for estimation of sulfadoxine residue level in poultry meat marketed in Chennai city. Of 102 poultry meat samples were analyzed, 16.7% sample had shown detectable levels of sulfadoxine and concentration varied between 1.03 and 23.8 ppb which were within limit RSD values at the three levels of fortification (5, 10, and 15ppb). A LC-MS/MS method was developed and validated for the determination of Tet^+^ residue in muscles samples in accordance with commission decision 2002/657/EC. Of ten muscles were examined for Tet^+^ residues where detection capability found in varied range between 122.2 and 137.6 μg/kg which depending on the analyte and the recoveries of all target compounds were 91.8-03.6%. The DL was measured between 109.0 and 119.8 μg [30]. Sopik *et al*. [31] have developed and validated HPLC-MS/MS multi residues method according to commission regulation 2002/657/EC to determine Tet^+^, Oxy-tet^+^ hydrochloride, Chlor-tet^+^ hydrochloride, and doxycycline hydrochloride with liquid-liquid extraction with trichloroacetic acid and McIlvaine buffer, followed by solid-phase extraction with HLB column was used to clean up the sample extract. LOD was measured 11.50, 9.96, 7.86, and 3.40 μg/kg, respectively, for Tet^+^, Oxy-tet^+^, Chlor-tet^+^, and doxycycline and overall recovery range 92.2%, 86.9%, 86.4%, and 78.9%, respectively, Tet^+^, Oxy-tet^+^, Chlor-tet^+^, and doxycycline, and the recovery of Tet^+^ ranged from 80.2 to 93.8% with RSD 7.3 %. In addition, an economic saving was also achieved by decreasing the run time to just 10 min, The LOQ 38.22, 33.20, 26.22, and 11.32 μg/kg, respectively, Tet^+^, Oxy-tet^+^, Chlor-tet^+^, and doxycycline [31]. In a new study a simple positive ion LC-MS/MS method was developed and validated as per 2002/657/EC, parameters such as system suitability, calibration curve, specificity, repeatability, recovery, laboratory reproducibility, DL, and DC. Linearity coefficient of determination was (R2) <0.99, LOD 17.93, 16.04, 19.67, 20.54, 20.52, and 18.98 for 4-epi-chlortet^+^, 4-epi Oxy-tet^+^, Chlor-tet^+^, 4-epi-tet^+^, Oxy-tet^+^, Tet^+^, respectively, and LOQ was 54.35, 48.63, 59.62, 62.26, 62.18, and 57.54 for 4-epi Chlor-tet^+^, Epi-Oxy-tet^+^, 4-epi-tet^+^, Chlor-tet^+^, Oxy-tet^+^, and Tet^+^, respectively. Recovery ranged measured between 83.07 and 115.58%, DL measured between 108.24 and 114.48 and DC was 116.48-129.61 [32]. Ribeiro *et al*. [33] developed and validated a method to determine Sulfa^+^, Tet^+^, and macrolides in honey sample, using LC-MS/MS according to Council Directive 657/EC/2002. Recoveries between 36 and 139% were obtained. Analyzed in three different 3 days concentrations ranging from 0 to 200% of the MRL, accuracy was between 89 and 113%, and intraday and interday precision with CV% (n=6) lower than 20%. LOQ for macrolides was 2.5 ng/g and for Sulfa^+^ and Tet^+^ was 5ng/g. The DC (CCβ) was between 15.8 and 36.3 ng/g, and DL (CCα) was between 12.9 and 28.1 ng/g. Von Eyken *et al*. [34] developed and validated HPLC-QTOF-MS for fast screening and quantification of veterinary drug residues in honey. The recoveries (103-119%), the linearity (R≥0.996), and the repeatability (RSD ≤7%) were satisfactory. This method allows detection of selected veterinary drug residues 20-100 times lower than regulatory limits, with accepted recoveries, linearity, and repeatability. Residues of tylosin A, tylosin B, sulfamethazine, and sulfadimethoxine were detected in 6, 9, 6, and 23% of sample, respectively, at level below the regulatory limit in Canada. Kivrak *et al*. [35] developed UPLC-ESI-MS/MS for simultaneous analysis of pharmaceuticals belong to three different classes Sulfa^+^, Amphenicols, and Tet^+^ in honey. LOD and LOQ of Sulfa^+^ group of antibiotics were ranged between 0.15-0.54 μg/kg and 0.26-0.90μg/kg for sulfacetamide and sulfisoxazole, respectively, A fast, time-effective, and simple sample preparation method was performed in approximately 35 min, and instrumental run time was only 8 min. LOQ was ranged from 0.24 to 0.58 μg/kg for epi-Tet+ and epi-Oxy-tet+, respectively. Elkhabeer *et al*. [36] developed an accurate analytical for the determination of 4 Tet^+^, Chl^+^, and 17 Sulfa^+^ residues in samples of chicken from different farms of Egypt. Of 60 samples were tested using LC-MS/MS. Instrument linearity was established using a multi-level calibration curve from 1 to 100 μg/L for Sulfa^+^ and Tet^+^ and from 0.1 to 20 μg/L for chl^+^; and the correlation coefficient was ≥0.995 for all compounds. Guidi *et al*. [37] developed and validated LC-ESI-MS/MS a sensitive, fast (15 min) and simple method for the screening of six classes of antibiotics (β-lac^+^, Aminoglycoside, Sulfa^+^, macrolides, tet^+^, and quinolones) in fish sample. Of 193 real fish samples were collected from aquaculture and analyzed where, 15% samples were found positive for enrofloxacin (quinolone). Matus *et al*. [38] were optimized and performed LC-MS/MS in flatfish samples for the determination of 60 compounds multi-residues. The Sample extraction was carried out by acetonitrile: water in ratio of 4:1 (v/v), with 10 mL volume. The sample treated by c18 and saturated hexane in 10 mL acetonitrile. Samples were precontracted by evaporation and finally reconstituted with mobile phase. HPLC-MS was validated as per (CAC/GL-71) codex guideline and used for sample screening. The results had coefficient variations of 1.6-22.1%, LOQ was 0.0005-0.005 μg/kg and recoveries of 73.2-115% and in fishery products. The proposed method can be applied to monitoring of real samples; there was no sample that exceeded the limit of quantification.

**Figure-3:**
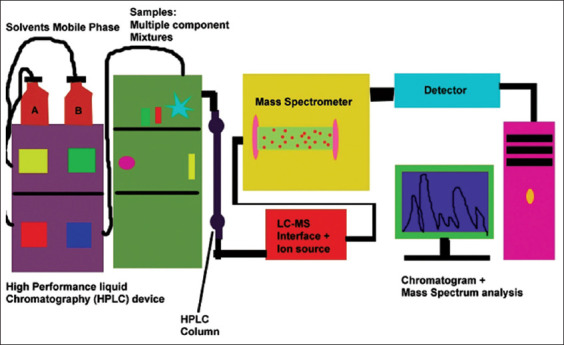
Work flow design of liquid chromatography mass spectrometry [Source: Figure prepared by Manthena Nava Bharath].

**Figure-4:**
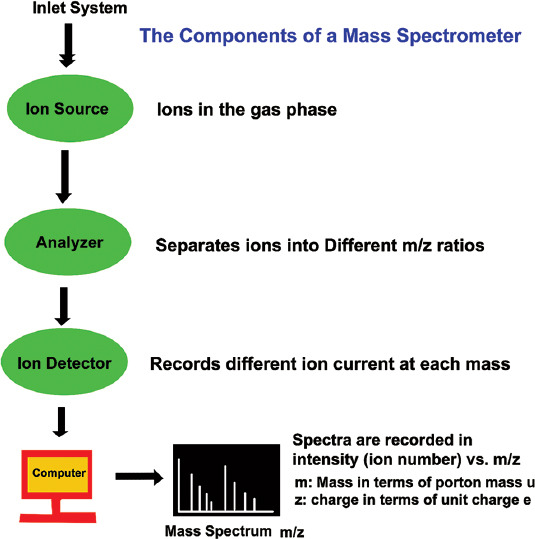
Graphical illustration of mass spectrometer [Source: Figure prepared by Manthena Nava Bharath].

Ultra-performance LC coupled to quadrupole time-of-flight MS (UPLC-QTOF-MS) is a very easy, multi residues, and multi-class analytical determination method which were developed for the screening, determination, and quantification of antibacterial/drugs in milk. In this study, of 90 veterinary drugs were determined from 20 classes, including macrolides, Sulfa^+^, lincomycin, quinolones, β-agonists, tet^+^, β-lac^+^, sedatives, sex hormones, β-receptor antagonists, nitroimidazole, glucocorticoid, benzimidazole, and nitrofurans with other analytes. The sample extraction method was used to extract the sample with acidifying acetonitrile with Quechers method. Parameters of validation were sensitivity, linearity, accuracy, and reproducibility. LOQ was calculated for these compounds present in milk ranging between 0.1 and 17.30 μg/kg. The repeatability and reproducibility were measured of 2.11-9.62% and 2.76-13.9%, respectively. The average recoveries measured between 72.62 and 122.2% with RSD (n=6) of 1.30 and 9.61% at three different concentrations levels. The range of correlation coefficient (R2) of 0.9973-0.9999 [39]. Britzi *et al*. [40] developed and validated LC-MS/MS method for simultaneous identification and quantification of eight nonsteroidal, Chl^+^ and anti-inflammatory drugs (NSAIDs) in bovine milk. This method is detecting and quantitating Tolfenamic, Carprofen, acid, 4-methyl aminoantipyrine, diclofenac, meloxicam, ibuprofen, Chl^+^, and phenylbutazone at their MRLs. The method accuracy was in the range of 89-108% and coefficients of variation of the interday precision assessment varied between 3 and 16%. Imamoglu and Oktem Olgun [41] developed and validated LC-MS/MS for multiclass and detection of ethyl acetate for the determination and quantification 187 total pesticide residues in milk and veterinary drugs. The average recoveries measured 75-120% with the RSD (n=18). This method was developed and validated as per criteria set in commission Decision 2002/657/EC and SANTE/11945/2015. The repeatability and reproducibility were measured between 2-13% and 6-16%, respectively. Schwaiger *et al*. [42] developed and validated Multi-Class UHPLC-MS/MS Method as per EC 657/2002 and simultaneous determined 30 substances from different groups of compounds (quinolones, lincosamides, macrolides, diamino-pyrimidine derivates and β-lac^+^, Tet^+^, and Sulfa^+^) in various kinds of dairy products. Erythromycin A and penicillin G are most problematic substances having high recovery rates and high standard deviations. Erythromycin A is not possible to determine in yogurt and curd. The recovery rates were measured 70 and 120% and repeatability was <20% for nearly all substances. Sulfathiazole and tilmicosin compounds were providing recovery rates up to 200% and high standard deviations up to 60% measured in mostly low spiked sample matrices. Ozdemir and Kahraman [43] developed a method for quick and confirmatory analysis of avermectins (doramectin, abamectin B1a, eprinomectinB1a, moxidectin, and ivermectin B1a) in bovine milk as per regulations 2002/657/EC requirements. The validation parameters were specificity, linearity, recovery, LOD, LOQ, DL, and DC. The LOQ ranged between 1.55 and 36.21 μg/kg, and LOD ranged between 1.17 μg/kg and 24.86 μg/kg, Mean recovery ranged between 78 and 111%, percent RSD <14%, decision limit (CCα), and detection capability (CC-β) ranged between 1.13-23.79 mg/kg and 1.21-26.32 mg/kg. HPLC coupled to high-resolution orbitrap MS was developed and validated by Pugajeva *et al*. [44] as per 2002/657/EC. They compared different sample preparation procedures and optimized for the detection of selected veterinary drugs in chicken, porcine, and bovine meat. In this method, most of the compounds in chicken meat, 123 compounds in porcine meat and 127 compounds in bovine meat could be quantified with RSD <30%, accuracy ranging from 70 to 120%. Delvotest SP was developed and standardized in 2009-2010 for determination and quantification of drug residues in raw milk in six different major regions of Kosovo. Raw milk was collected from individual farms and milk collection points during 2009-2010. Of 1734 samples examined with Delvotest SP contained possible drug residues (5.12% and 7.51% of samples from 2009 and 2010, respectively). In the present study, Delvotest SP assay and an enzyme-linked receptor-binding assay (SNAP) were used for screening. Only the new SNAP β-lac^+^ test detected residues in 40 out of 52 samples in 2009 and 54 out of 54 suspect samples in 2010. All suspected samples were further analyzed by three distinct SNAP specific for β-lac^+^, Tet^+^, and Sulfa^+^ [45].

## Ion Source

It’s a device that generates atomic and molecular ions for particle accelerators, ion implanters, mass spectrometers, optical emission spectrometers and ion engines. There are three types of ionization techniques such as ESI, APCI, and APPI.

## Electrospray Ionization (ESI)

**Figure-5:**
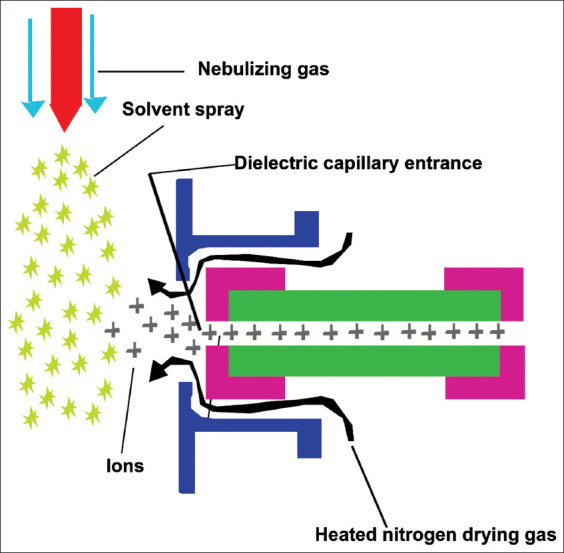
Graphical representation of electrospray ionization [Source: Figure prepared by Manthena Nava Bharath].

The ESI is a soft ionization technique, this technique is applicable for thermally unstable compounds such as polar and mid polar in nature. This technique is mostly using because of its cost-effectiveness. In this ionization technique, the mobile phase comes in a liquid form and nitrogen gas as nebulizer gives spray form and convert into ions with the apply high voltage in thin capillary and the nebulizer gas as nitrogen and due to high voltage, the big droplet of the ions becomes smaller and slowly convert into the ions due to the replication of the same nature of the ions likes positive-positive. In the ESI technique, the mobile phase is an organic solvent such as methanol and acetonitrile. The ion source is a high vacuum chamber where the vapor of the solvent becomes the charged ions and entered into analyzer chamber of the MS (Figure-5). The ESI technique is applicable to pesticide residues, mycotoxins, and antibiotics residues.

## Atmospheric Pressure Chemical Ionization (APCI)

**Figure-6:**
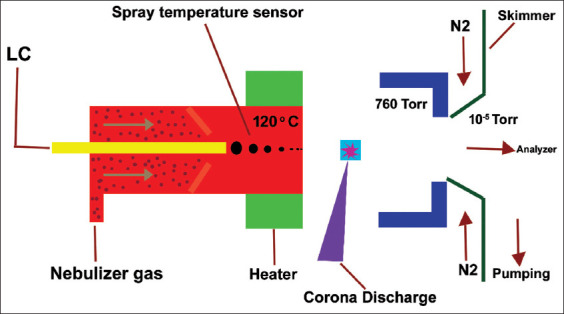
Run diagram of atmospheric pressure chemical ionization [Source: Figure prepared by Manthena Nava Bharath].

The APCI is the technique applicable to non-polar compounds. In this technique, the liquid mobile phase pass through and evaporated due to high temperature in applying in heater blocks and ionization in the corona discharge, the ions are generated and entering into the mass analyzer. It is an applicable thermally stable compound. In this ionization, the electron from the 63 Ni beta emitter and these used gases as nitrogen or air. The heated nebulizer produced the ions at atmospheric pressure in a chemical reaction. The APCI technique is sensitive, robust, and reliable, and its generated ions greater than ESI (Figure-6).

## Atmospheric Pressure Photoionization (APPI)

**Figure-7:**
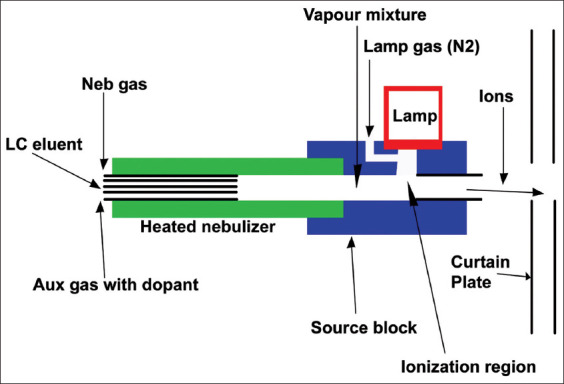
Image of atmospheric pressure photoionization [Source: Figure prepared by Manthena Nava Bharath].

APPI is a soft ionization technique for LC-MS that uses photochemical action, which helps to ionize the analytes in the gas phase. It also facilitates the analytical detection of weakly polar and non-polar compounds by mass spectrometry. It is a very sensitive technique compares to ESI and APCI (Figure-7).

## Conclusion

The importance of all techniques based on their applications. Here, we are systematically described as ELISA, HPLC, and LCMS techniques. The ELISA is more suitable for antigen-antibody interaction. ELISA is based on kit-based method; it is a semi-quantitative technique, some time we analyzing the sample present or absent and quantitative as well. Basically, ELISA has been prominently used in diagnostic labs. However, with the help of HPLC technique, we can analyze the sample quantitatively. This technique is providing more confidence on this basis of unknown sample versus know using standard. The interpretation is matching of sample and standard on same wavelength, same RT, there are many applications such as Pharmaceutical, Food industries, and Research. If we look more advancement method, LCMS is a very sophisticated technique based on m/z ratio values proving confidence as compared to HPLC. It gives quantitative as well as confirmation. It is also categorized into three phases such as ESI, APCI, and APPI. The ESI technique is more popular and suitable for polar and mid-polar compounds such as antibiotic residue and pesticide residues. In APPI is applicable for weekly polar and non-polar compounds and its soft ionization technique. Hence, determination of various drug residues using new high throughput technologies will give the possibility of automation, reduced time to obtain the result, better sensitivity and specificity and reduced error probability in detection capabilities will help in better safety assurance, standard and quality of various animal origin food products such as milk and meat.

## Authors’ Contributions

JKP and VG: Manuscript writing. KKC: Manuscript writing and revision. MNB: Figure preparation. All authors have read and approved the final manuscript.

## References

[1] Nunes K.S, Assalin M.R, Vallim J.H, Jonsson C.M, Queiroz S.C, Reyes F.G (2018). Multiresidue method for quantification of sulfonamides and trimethoprim in tilapia fillet by liquid chromatography coupled to quadrupole time-of-flight mass spectrometry using QuEChERS for sample preparation. J. Anal. Methods Chem.

[2] Elbagory A.M, Yasin N.A, Algazar E.A (2016). Effect of various cooking methods on some antibacterial residues in imported and local frozen dressed broilers and their giblets in Egypt. Nutr. Food Technol.

[3] Lan C, Yin D, Yang Z, Zhao W, Chen Y, Zhang W, Zhang S (2019). Determination of six macrolide antibiotics in chicken sample by liquid chromatography-tandem mass spectrometry-based on solid-phase extraction. J. Anal. Methods Chem.

[4] Maddaleno A, Pokrant E, Yanten F, San Martin B, Cornejo J (2019). Implementation and validation of an analytical method for lincomycin determination in feathers and edible tissues of broiler chickens by liquid chromatography-tandem mass spectrometry. J. Anal. Methods Chem.

[5] Furusawa N (2018). Quantification of residual thiabendazole and its metabolites, 5-hydroxythiabendazole, in cow's milk using pipetting sample preparation with water eluent and water mobile phase HPLC coupled diode array:Method development and validation. Int. J. Chem. Sci.

[6] Jedziniak P, Olejnik M, Pietruk K, Protasiuk E, Szprengier-Juszkiewicz T, Żmudzki J (2016). Simultaneous determination of residues of non-steroidal anti-inflammatory drugs and glucocorticosteroids in animal muscle by liquid chromatography-tandem mass spectrometry. Food Anal. Methods.

[7] Tao Y, Xie S, Zhu Y, Chen D, Pan Y, Wang X, Liu Z, Huang L, Peng D, Yuan Z (2018). Analysis of major components of bacitracin, colistin and virginiamycin in feed using matrix solid-phase dispersion extraction by liquid chromatography-electrospray ionization tandem mass spectrometry. J. Chromatogr. Sci.

[8] Reddy B.S, Yadlapalli S, Rao Y.S, Prasad P.R, Prabhakar K, Sreedhar N.Y (2018). LC/ESI/MS/MS method development and validation for the determination of sulfonamide antibiotics residues in shrimp sample. J. Ultra Chem.

[9] Bion C, Beck Henzelin A, Qu Y, Pizzocri G, Bolzoni G, Buffoli E (2016). Analysis of 27 antibiotic residues in raw cow's milk and milk-based products validation of Delvotest®T Part A Chemistry, analysis, control, exposure and risk assessment. Food Addit. Contam. Part A Chem. Anal. Control Expo Risk Assess 2016.

[10] Layada S, Benouareth D.E, Coucke W, Andjelkovic M (2016). Assessment of antibiotic residues in commercial and farm milk collected in the region of Guelma (Algeria). Int. J. Food Contam.

[11] Romero T, Althaus R, Moya V.J, del Carmen Beltrán M, Reybroeck W, Molina M.P (2017). Albendazole residues in goat's milk:Interferences in microbial inhibitor tests used to detect antibiotics in milk. J. Food Drug Anal.

[12] Kim H.J, Jeong M.H, Park H.J, Kim W.C, Kim J.E (2016). Development of an immunoaffinity chromatography and HPLC-UV method for determination of 16 sulfonamides in feed. Food Chem.

[13] Patyra E, Przeniosło-Siwczyńska M, Kwiatek K (2019). Determination of sulfonamides in feeds by high-performance liquid chromatography after fluorescamine precolumn derivatization. Molecules.

[14] Kellnerová E, Navrátilová P, Borkovcová I (2015). Effect of pasteurization on the residues of tetracyclines in milk. Acta Vet. Brno.

[15] Saleh S.M.K, Mussaed A.M, Al-Hariri F.M (2016). Determination of tetracycline and oxytetracycline residues in honey by high-performance liquid chromatography. J. Agric. Sci. Technol.

[16] Chauhan S.L, Priyanka G.S, Jadhav V.J (2019). Determination of tetracycline residues in milk by high-performance liquid chromatography. Int. J. Curr. Microbiol. Appl. Sci.

[17] Marinou E, Samanidou V.F, Papadoyannis I.N (2019). Development of a high-pressure liquid chromatography with diode array detection method for the determination of four tetracycline residues in milk by using QuEChERS dispersive extraction. Separations.

[18] Prado C.K, Ferreira F.D, Bando E, Junior M.M (2017). In-house validation for multi-residue analysis of tetracycline in cow milk by HPLC with UV detection. Semin. Cienc. Agrar.

[19] Shaltout F.A, Shatter M.A.E, Fehim H.M (2019). Studies on antibiotics residues in beef and effect of cooking and freezing on antibiotics residues beef samples. Sch. J. Food Nutr.

[20] Mostafa A.E, Salam R.A.A, Hadad G.M, Eissa I.A (2017). Simultaneous determination of selected veterinary antibiotics in Nile tilapia (*Orechromis niloticus*) and water samples by HPLC/UV and LC-MS/MS. RSC Adv.

[21] Darko G, Borquaye L.S, Acheampong A, Oppong K (2017). Veterinary antibiotics in dairy products from Kumasi, Ghana. Cogent Chem.

[22] Kurjogi M, Mohammad Y.H.I, Alghamdi S, Abdelrahman M, Satapute P, Jogaiah S (2019). Detection and determination of stability of the antibiotic residues in cow's milk. PLoS One.

[23] Orwa J.D, Matofari J.W, Muliro P.S, Lamuka P (2017). Assessment of sulphonamides and tetracyclines antibiotic residue contaminants in rural and peri-urban dairy value chains in Kenya. Int. J. Food Contam.

[24] Hamamoto K, Mizuno Y (2017). LC-MS/MS measurement of ampicillin residue in chicken tissues at 2 days after in-feed administration. J. Vet. Med. Sci.

[25] Jedziniak P, Olejnik M, Rola J.G, Szprengier-Juszkiewicz T (2015). Anthelmintic residues in goat and sheep dairy products. Bull. Vet. Inst. Pulawy.

[26] Chen X, Peng S, Liu C, Zou X, Ke Y, Jiang W (2019). Development of an indirect competitive enzyme-linked immunosorbent assay for detecting flunixin and 5-hydroxyflunixin residues in bovine muscle and milk. Food Agric. Immunol.

[27] Unsal I.A, Tasan M, Gokcen T, Goren A.C (2018). Determination of sulfonamides in milk by ID-LC-MS/MS. J. Chem. Metrol.

[28] Wang J, Hu Q, Li P, Fang Y, Yang W, Ma N, Pei F (2019). Comparison of three different lipid removal clean-up techniques prior to the analysis of sulfonamide drug residues in porcine tissues. Food Sci. Nutr.

[29] Govind V, Babu R.N, Rao V.A, Sriram P, Senthil T.M.A (2018). Determination of sulfadoxine residues in poultry meat by liquid chromatography and tandem mass spectrometry. J. Entomol. Zool. Stud.

[30] Gajda A, Posyniak A (2015). Liquid chromatography-tandem mass spectrometry method for the determination of ten tetracycline residues in muscle samples. Bull. Vet. Inst. Pulawy.

[31] Šopík T, Vydrová L, Zálešáková L, Buňka F (2016). Development of a method for analysis of tetracycline residues in cow's milk by liquid chromatography/tandem mass spectrometry. Int. J. Food Sci. Technol.

[32] Reddy B.S, Sudhakar Y, Rao Y.S, Reddyprasad P, Sreedhar N.Y (2017). LC-MS/MS method development and validation to determine three tetracyclines and their epimers in shrimp samples. Orient. J. Chem.

[33] Ribeiro C.B, Martins M.T, Jank L, Barreto F, Hoff R.B, Arsand J.B (2018). Development and validation of a simple and fast method for sulfonamides, tetracyclines and macrolides in honey using LC-MS/MS. Drug Anal. Res.

[34] Von Eyken A, Furlong D, Arooni S, Butterworth F, Roy J.F, Zweigenbaum J, Bayen S (2019). Direct injection high-performance liquid chromatography coupled to data-independent acquisition mass spectrometry for the screening of antibiotics in honey. J. Food Drug Anal.

[35] Kivrak I, Kivrak Ş, Harmandar M (2016). Development of a rapid method for the determination of antibiotic residues in honey using UPLC-ESI-MS/MS. Food Sci. Technol.

[36] Elkhabeer M.A, Gouda G.A.R, Ryad L, Souaya E.R (2020). Analytical method optimization and determination of sulfonamides, chloramphenicol and tetracyclines drug residues in chicken meat across Egypt. J. Food Process. Technol.

[37] Guidi L.R, Santos F.A, Ribeiro A.C.S, Fernandes C, Silva L.H, Gloria M.B.A (2017). A simple, fast and sensitive screening LC-ESI-MS/MS method for antibiotics in fish. Talanta.

[38] Matus J.L, Boison J.O (2016). A multi?residue method for 17 anticoccidial drugs and ractopamine in animal tissues by liquid chromatography?tandem mass spectrometry and time?of?flight mass spectrometry. Drug Test. Anal.

[39] Zhang Y, Li X, Liu X, Zhang J, Cao Y, Shi Z, Sun H (2015). Multi-class, multi-residue analysis of trace veterinary drugs in milk by rapid screening and quantification using ultra-performance liquid chromatography-quadrupole time-of-flight mass spectrometry. J. Dairy Sci.

[40] Britzi M, Schwartsburd F (2019). Development and validation of a high-throughput method for the determination of eight non-steroidal anti-inflammatory drugs and chloramphenicol in milk, using liquid chromatography-tandem mass Spectroscopy. Int. J. Anal. Bioanal. Methods.

[41] Imamoglu H, Olgun E.O (2016). Analysis of veterinary drug and pesticide residues using the ethyl acetate multi-class/multi-residue method in milk by liquid chromatography-tandem mass spectrometry. J. Anal. Methods Chem.

[42] Schwaiger B, König J, Lesueur C (2018). Development and validation of a multi-class UHPLC-MS/MS method for determination of antibiotic residues in dairy products. Food Anal. Methods.

[43] Ozdemir N, Kahraman T (2016). Rapid confirmatory analysis of avermectin residues in milk by liquid chromatography-tandem mass spectrometry. J. Food Drug Anal.

[44] Pugajeva I, Ikkere L.E, Judjallo E, Bartkevics V (2019). Determination of residues and metabolites of more than 140 pharmacologically active substances in meat by liquid chromatography coupled to high-resolution Orbitrap mass spectrometry. J. Pharm. Biomed. Anal.

[45] Rama A, Lucatello L, Benetti C, Galina G, Bajraktari D (2017). Assessment of antibacterial drug residues in milk for consumption in Kosovo. J. Food Drug Anal.

